# Hyaluronic acid as a pulpotomy material in primary molars: an up to 30 months retrospective study

**DOI:** 10.1186/s12903-024-04405-4

**Published:** 2024-06-12

**Authors:** Batın Ilgıt Sezgin, Gökce Cicek Ildes Sezgin, Özge Koyuncu, Ali Mentes

**Affiliations:** 1 Department of Paediatric Dentistry, Faculty of Dentistry, Istanbul Galata University, Istanbul, Türkiye 34430 Turkey; 2 Private practice, Istanbul, Türkiye 34843 Turkey; 3 Department of Paediatric Dentistry, Faculty of Dentistry, Marmara University, Istanbul, Türkiye 34854 Turkey

**Keywords:** Pulpotomy, Primary molars, Hyaluronic acid, Formocresol, Ferric sulphate, Retrospective study

## Abstract

**Background:**

The aim of this retrospective study was to determine the long-term clinical and radiographic success of our previous randomized clinical trial and to compare the success of hyaluronic acid, with the widely used formocresol and ferric sulphate agents.

**Methods:**

This retrospective study is the extension of the 1-year survey of our randomized clinical trial that had compared the effectiveness of a hyaluronic acid pulpotomy over formocresol and ferric sulphate pulpotomies and included clinical and radiographic evaluations with a follow-up period of over 24 months for 44 children who applied to our clinic between May 2019 and September 2019. Long-term clinical and radiographic data were obtained from the periodic files of our department, wherein each tooth’s file was examined to identify any clinical and radiographic findings. Descriptive statistics and Pearson’s chi-square tests were used to evaluate the data. Statistical significance was considered as *p* < 0.05.

**Results:**

The clinical and radiographic success rates of the hyaluronic acid, formocresol, and ferric sulphate groups were not statistically different at > 24 months. None of the teeth in the hyaluronic acid group showed any clinical findings at > 24 months.

**Conclusions:**

Hyaluronic acid pulpotomies exhibited comparable success rates to formocresol and ferric sulphate materials spanning over 24 months examinations. Because of convenient accessibility and applicability of hyaluronic acid, it may be recommended as a promising alternative medicament for pulpotomy treatments of primary molars. However, further long-term follow-up human studies are needed to better understand the effect of hyaluronic acid on the dental pulp of human primary molars.

## Background

Primary molar pulpotomy is one of the vital pulp treatments aimed at removing inflamed or infected pulp tissue from the coronal pulp chamber, while simultaneously preserving the vitality of the remaining radicular pulp tissue through the application of various medicaments. Indicated in cases of normal or reversible pulpitis, pulpotomy is essential during the removal of caries-induced pulp exposure or post-traumatic scenarios, with the requirement that there are no radiographic indications of infection or pathological resorption in the relevant tooth [1]. The effectiveness and success of pulpotomy vary depending on the material used, and throughout history, there has always been a quest to find the ideal and perfect pulpotomy agent and/or technique. The expectations from an ideal pulpotomy agent include controlling existing bacterial infection, demonstrating biocompatibility with remaining tissues, inducing hard tissue formation without affecting physiological root resorption, and being economically feasible [2]. A myriad of agents or techniques with different mechanisms of action has been applied to the radicular pulp tissue in the pursuit of finding the ideal pulpotomy treatment material. Among these agents or techniques include formocresol (FC), diluted formocresol (DFC), glutaraldehyde (GA), ferric sulphate (FS), calcium hydroxide (CH), mineral trioxide aggregate (MTA), biodentine (BD), zinc oxide eugenol (ZOE), sodium hypochlorite (NaOCl), and non-pharmacotherapeutic methods such as electrocautery and lasers [2].

In their literature review, Bossù et al. [3] reported that MTA, BD, and FS have consistently shown favorable clinical outcomes over time. They declared that these materials can be safely utilized in the pulpotomy treatment of primary molars. However, despite the clinical success of FC, they emphasized the necessity to replace it with other materials due to its potential cytotoxic and carcinogenic effects. In the 2017 publication of the American Academy of Pediatric Dentistry (AAPD) guidelines, it is recommended that for primary teeth expected to remain in the oral cavity for 24 months or longer, only MTA and FC are suggested for pulpotomy treatment. Recommendations were made against the use of CH, while the use of FS, NaOCl, lasers, and tricalcium silicate materials or techniques were conditionally suggested only at a weak level of evidence [4]. In the literature, the presence of conflicting results regarding pulpotomy materials or techniques used up to the present day, coupled with the absence of a consensus on the ideal pulpotomy material, has led to an ongoing quest for new materials with the development of technology.

Hyaluronic acid (hyaluronan, HA) is present in vertebrate tissues as a crucial component of the extracellular matrix (ECM). It exhibits a straightforward covalent structure composed of alternating β-D-glucuronate and *N*-acetyl β-D-glucosamine sugars [5]. In most tissues, native HA possesses a high molecular mass of 1–10 million Da with extended molecular lengths of 2–20 μm [6–9]. Despite its simple structure, HA is an exceptionally versatile macromolecule. Its biophysical properties confer upon HA functions that impact the hydration and biomechanical properties of various tissues, particularly those of the vitreous humor in the eye, the synovial joint fluid, and the dermis [10]. Additionally, HA also interacts with extracellular macromolecules and HA-binding proteoglycans, such as versican and aggrecan, crucial for the assembly of ECMs and of pericellular glycocalyces, which can function as protective cellular barriers and are essential for the assembly and structure of various tissues [10–13]. Physiological functions of HA are mediated by molecular interactions with CD44 and RHAMM and other HA-binding proteins [11]. HA-rich glycocalyces anchored on cell surfaces by CD44 and RHAMM can trigger intracellular signaling pathways [11, 14, 15], which can prompt gene expression associated with cell-cell adhesion, cell spatial orientation and trafficking, cell growth and differentiation, inflammation [9], wound healing and tissue remodeling [9, 16, 17], tissue morphogenesis [9, 18]. Successful morphogenesis also depends on the physical properties of HA, in addition to the signaling events induced by HA-CD44 and/or HA-RHAMM interactions. In the course of embryogenesis, HA encourage proliferation and migration of undifferentiated stem cells to sites of organ development [19].

Due to HA being a natural and widely distributed component of the tissue ECM and its numerous active roles at the cellular and tissue levels as mentioned above, it is made use of in various branch of dentistry, including the treatment of gingivitis [20], chronic periodontitis [21], peri-implantitis [22], and alveolar osteitis [23], management of wound healing after free gingival graft [24], control of symptoms such as pain, swelling, and trismus that may occur after tooth extraction [25], healing of recurrent oral ulcers [26], and management of teething symptoms [27]. HA is also used in studies involving human dental pulp stem cells (hDPSCs) with the aim of providing a new approach to regenerative endodontic treatments [28].

In our previous randomized clinical trial (RCT), encompassing a 1-year follow-up, we found that HA, FC, and FS agents used in pulpotomy treatments of human primary molar teeth yielded comparable success rates both clinically and radiographically [29].

Building upon these outcomes, the aim of this retrospective study was to determine the long-term clinical and radiographic success of our previous RCT [29] and to compare the success of HA, a promising agent for pulpotomy treatment, with the widely used FC and FS agents.

## Methods

This retrospective study is the extension of the 1-year survey of our RCT study that had compared the effectiveness of a HA pulpotomy over FC and FS pulpotomies [29] and included clinical and radiographic evaluations with a follow-up period of over 24 months for the 128 teeth of the 44 children (27 girls, 17 boys; aged initially between 5 and 9) who applied to the Department of Paediatric Dentistry at Marmara University between May 2019 and September 2019 and underwent pulpotomy treatments using HA, FC, and FS materials. This study was performed in line with the principles of the Declaration of Helsinki. The waiver of the requirement to obtain informed consent from patients, as well as the approval of the study protocol, was provided by the Clinical Research Ethics Committee, Faculty of Medicine at Marmara University (No: 12.01.2024.56).

In the previous study, comprehensive information had been provided regarding the registration of the study as a randomized clinical trial, the sample size calculation performed through power analysis, the selection criteria for children and teeth included in the study both clinically and radiographically, treatment procedures, duration of follow-up periods, and the clinical and radiographic success criteria considered during these periods [29].

Briefly, at the beginning, all children undergoing treatment, retrospectively examined, were those who did not require sedation or general anaesthesia, demonstrated good compliance according to the Frankl Behavior Rating Scale, and had no allergies or systemic diseases. In the present study, the initial clinical selection criteria for the long-term evaluation of primary molars were as follows: having deep carious lesions approaching or reaching the pulp; absence of spontaneous, continuous, or nocturnal pain; physiological or pathological mobility; abscess and/or fistula formation; showing no tenderness to palpation or percussion; and the ability to be restored with stainless steel crowns or composite filling material after treatment. Radiographic selection criteria were as follows: teeth without physiological root resorption or exceeding one-third of the root; without internal/external pathological root resorption; not showing radiolucency in periapical or furcation regions; and without periodontal ligament widening. The treatments of all teeth meeting the clinical and radiographic selection criteria were performed by the same experienced paediatric dentist (G.C.I.S.).

For the treatment procedure, although detailed information had been provided in 1-year follow-up RCT [29], we briefly touched upon it here. Primary molars were initially randomly allocated to three groups: 0.5% Hyaluronic Acid Gel group (Gengigel®Teething, Ricerfarma, Italy), Formocresol group (Prevest DenPro®, India), and 20% Ferric Sulphate group (ViscoStat™, Ultradent Products Inc.). The standardized clinical treatment procedure for all primary molars was local anaesthesia followed by rubber dam isolation (Roeko Dental Dam®, Coltene Whaledent), the removal of the roof of the pulp chamber, amputation of the pulp tissue within the pulp chamber until the pulp canal orifices were clearly visible. Subsequently, the pulp chamber was irrigated with a saline solution, sterile cotton pellets moistened with saline solution were applied directly to the pulp canal orifices with gentle pressure to achieve hemostasis, and they were left in place for 4 min. Once successful hemostasis was achieved, a cotton pellet saturated with each treatment material was applied to the pulp canal orifices for appropriate durations (15 s for FS, 2 min for FC, and an estimated 2 min for HA, comparable to FC). Following the removal of excess materials from the pulp chamber by using a dry and sterile cotton pellet, the pulp chambers were filled with ZOE cement (ZOE-Kalzinol, Dentsply). Finally, the teeth were restored in the same appointment with composite filling material (GC Gradia Direct Posterior, Japan) in the presence of occlusal cavities and stainless steel crowns (SSCs) (3 M™, Unitek™, USA) in the presence of proximal cavities. Initial, 1st, 3rd, 6th, and 12th month clinical and radiographic evaluations were completed in the previous study [29].

In this retrospective study, the long-term clinical and radiographic documents were detected from the periodic files of our department by the same paediatric dentist (Ö.K). The files for each tooth were investigated for the presence of one or more of the following clinical findings: spontaneous pain, swelling, percussion and/or palpation tenderness, pathological mobility, abscess, fistula formation, exfoliation or extraction, while x-rays were detected for the clarity of the treated tooth and surrounding bone tissue. Radiographically, the presence of one or more of the following findings was considered failure: periapical or furcal radiolucency, internal or external root resorption, loss of lamina dura, and periodontal ligament widening. Pulp canal obliteration (PCO) was not considered as a failure.

The analysis of the collected clinical data and obtained radiographs from the files was performed by two calibrated experienced paediatric dentists (A.M., B.I.S.). In cases of disagreement between the two reviewers, reassessments were conducted to reach a consensus, and if an agreement could not be reached, the assessment with the more unfavorable outcome was considered. The intra- and inter-rater reliability were calculated using Cohen’s unweighted kappa statistic (Intra and inter-rater reliability; 0.90, and 0.88 respectively).

SPSS 22.0 statistical software (IBM Inc., Chicago, Ill, USA) was employed for the analysis of the data. Descriptive statistics and Pearson’s chi-square tests were used to evaluate the data. Statistical significance was considered as *p* < 0.05.

## Results

**Fig. 1:**
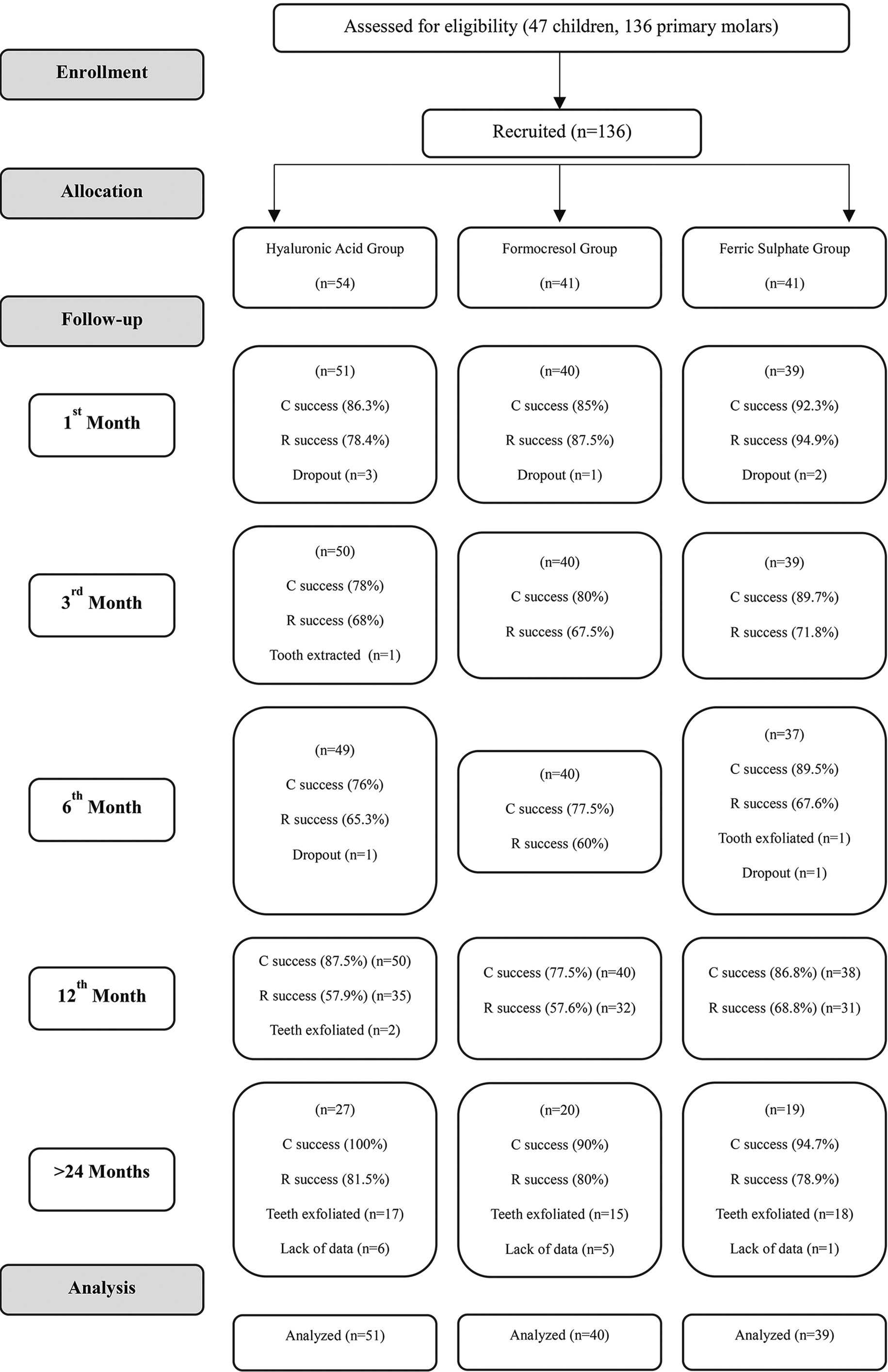
Flowchart depicting the pulpotomized teeth and their clinical/radiographic success rates over 24 months (C: Clinical; R: Radiographic)

The retrospective follow-up data, comprising clinical and radiographic records, were available for the 116 teeth (44 HA, 35 FC, 37 FS) belonging to the initial 41 out of 44 children whose treatments had been completed. Data from 12 teeth (6 HA, 5 FC, 1 FS) in 3 children were missing. In all treatment groups, the mean duration of follow-up control visit detected was 28.21 ± 1.36 months with a minimum of 25 months and a maximum of 30 months. As expected, 50 teeth (17 HA, 15 FC, 18 FS) had exfoliated before the examination, but there was no statistically significant difference between the groups (*p* = 0.973). Thus, records from 66 teeth (27 HA, 20 FC, 19 FS) were available for final analysis (Fig. 1).

The clinical success rates of the HA, FC, and FS groups were not statistically different at > 24 months (*p* = 0.163). None of the teeth exhibited spontaneous pain, swelling, palpation tenderness, abscess, or fistula formation in all three groups at > 24 months. In the FS group, one tooth (5.3%) was recorded as sensitive to percussion, and exhibited pathological mobility, whereas two teeth from the FC group (10.0%) were noted to have pathological mobility at > 24 months. None of the teeth in the HA group showed any clinical findings at > 24 months (100% clinical success) (Table 1).

**Fig. 2:**
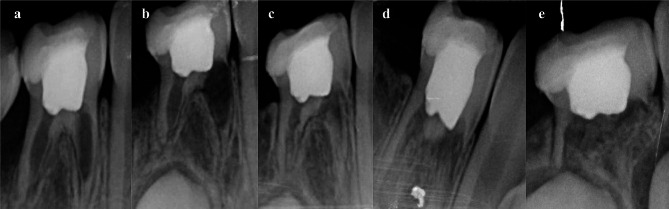
Severe internal root resorption of the primary mandibular right first molar with Formocresol. (**a**) 1st-month, (**b**) 3rd-month, (**c**) 6th-month, (**d**) 12th-month, (**e**) 27th-month

**Fig. 3:**
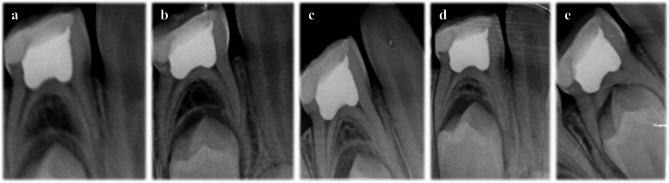
No pathological radiographic finding of the primary mandibular right first molar with Hyaluronic acid until its physiological exfoliation time. (**a**) 1st-month, (**b**) 3rd-month, (**c**) 6th-month, (**d**) 12th-month, (**e**) 26th-month

The radiographic success rates of the HA, FC, and FS groups were not statistically different at > 24 months (*p* = 0.977). The most common pathological radiographic findings were internal root resorption (3 HA-11.1%; 4 FC-20%; 2 FS-10.5%)(Fig. 2), followed by furcal radiolucency (3 HA-11.1%; 2 FS-10.5%). The most prevalent non-pathological radiographic features were periapical resorption (23 HA-85.2%; 14 FC-70%; 16 FS-84.2%), followed by PCOs (6 HA-22.2%; 4 FC-20%; 6 FS-31.6%) at > 24 months (Table 1)(Fig. 3). These subsequent findings were not deemed as a failure in the study.

**Table 1:** The number and percentage of clinical and radiographic findings of 3 study groups at > 24 months

	Hyaluronic Acid *n* = 27		Formocresol *n* = 20		Ferric Sulphate *n* = 19		*P* value
**Clinical Findings**							
Swelling	**n** 0	**%** 0.0%	**n** 0	**%** 0.0%	**n** 0	**%** 0.0%	-
Percussion	0	0.0%	0	0.0%	1	5.3%	0.282^‡^
Palpation	0	0.0%	0	0.0%	0	0.0%	-
Mobility	0	0.0%	2	10.0%	1	5.3%	0.168^‡^
Abscess	0	0.0%	0	0.0%	0	0.0%	-
Fistula	0	0.0%	0	0.0%	0	0.0%	-
**Radiographic Findings**							
Periapical radiolucency	**n** 0	**%** 0.0%	**n** 0	**%** 0.0%	**n** 0	**%** 0.0%	-
Furcal radiolucency	3	11.1%	0	0.0%	2	10.5%	0.150^‡^
Internal root resorption	3	11.1%	4	20.0%	2	10.5%	0.626^‡^
External root resorption	1	3.7%	0	0.0%	1	5.3%	0.464^‡^
Loss of lamina dura	0	0.0%	0	0.0%	0	0.0%	-
Periodontal ligament widening	0	0.0%	0	0.0%	0	0.0%	-
Pulp canal obliteration	6	22.2%	4	20.0%	6	31.6%	0.674^‡^
Periapical resorption	23	85.2%	14	70.0%	16	84.2%	0.400^‡^

^**‡**^ **Pearson’s Chi-Square Test**

## Discussion

Primary molar pulpotomy, one of the frequently utilized treatments for deep caries in children, still requires easily obtainable, affordable, and biocompatible materials. Analyses conducted highlight the absence of evidence to support material selection for these treatments [30]. Our study indicated that hyaluronic acid could be an alternative material with a high success rate in the long-term success of these treatments. The European Academy of Paediatric Dentistry (EAPD) [31], as outlined in its 2022 policy document, reported the high success rates of pulpotomy treatments, recommending them as an effective approach for managing deep caries in primary teeth. In the present study, we found that HA demonstrated similarly high success rates, both clinically and radiographically, during the long-term follow-up of pulpotomy treatments in human primary molar teeth. The policy document also highlighted that when employed as pulpotomy medicaments, FC, FS, and MTA exhibit comparable success rates; nevertheless, due to concerns regarding the potential toxic effects of FC, clinicians are recommended to use alternative medicaments like FS and MTA, which offer similar results. In this study, FC and FS materials were used as the control group, and it stood out as the first to demonstrate the long-term clinical and radiographic follow-up of HA in pulpotomy treatment of human primary molars, comparing its success with that of FC and FS materials.

A most recent network meta-analysis study by Guo et al. [30] included 43 randomized controlled trials, aiming to determine the comparative effectiveness of FC, FS, NaOCl, CH, MTA, BD, and laser pulpotomy treatments in primary molars. Upon analysis of studies reporting 12th-month success rates, it was observed that both clinically and radiographically, MTA, BD, and laser pulpotomy treatments were the most successful, with FC treatments having statistically comparable but superior values compared to FS treatments both clinically and radiographically. However, as a limitation of this network meta-analysis, it was emphasized that clinical and radiographic assessments were only conducted at 6th and 12th months, and the necessity to investigate long-term effectiveness when a sufficient number of appropriate studies are available was highlighted.

Huth et al. [32] stated in their study that while no statistically significant superiority was observed after 3 years, FS treatments demonstrated a better outcome in terms of both clinical and overall success rates compared to FC treatments. No statistically significant differences were reported between the groups (gray MTA, DFC) in terms of clinical success during any follow-up period in the study conducted by Sushynski et al. [33].

In the study conducted by Sonmez et al. [34], after a 2-year clinical and radiographic follow-up, it was reported that the number of radiographic findings was significantly higher than the number of clinical findings. At the end of 24 months, the total success rates for FC, FS, CH, and MTA were reported as 76.9%, 73.3%, 46.1%, and 66.6%, respectively, with no significant difference detected among the groups. Noorollahian [35] conducted pulpotomy treatments and stated that the teeth treated with 1:5 DFC showed no clinical or radiographic findings at any follow-up period, while in the white MTA group, one tooth each demonstrated clinical success but radiographically showed furcal radiolucency at the 12th and 24th month follow-ups. Erdem et al. [36] declared that the overall success rates had decreased in all groups (MTA, FS, FC, and ZOE) at the 24th-month follow-up compared to the 12th-month follow-up. Statistically significant differences were observed between the MTA and ZOE groups at the 24th-month follow-up, while no significant differences were reported among the other groups. Nematollahi et al. [37] carried out partial pulpotomy treatments using MTA agent and pulpotomy treatments of primary molars using FC agent, and noted that in the FC treatment group, clinical and radiographic success rates were 100% at the 6th and 12th-month follow-ups, while at the 24th-month follow-up, clinical success remained at 100%, with radiographic and total success reported as 95.2%. Petel et al. [38] reported that in their pulpotomy treatments of primary molars using pure Portland cement and FC agents, teeth treated with pure Portland cement showed 100% success clinically and radiographically throughout all follow-up periods. At the 48-month follow-up, the FC treatment group demonstrated a total success rate of 91.1%.

In our previous study [29], only 2 teeth in the HA group and 1 tooth in the FS group had exfoliated by the end of the first year. In our long-term examination, although there was no statistically significant difference between them, it relatively increased in all three groups. Consistent with our findings, Sushynski et al. [33], Noorollahian [35], and Petel et al. [38], with a minimum follow-up period of 2 years, similarly reported a significant reduction in the number of initially treated teeth by the final follow-up period. The mean age of the children included in our study was slightly higher than that reported in the aforementioned studies, and due to the treatment of 67 first primary molars out of the 130 primary molars treated (51.5%) [29], we believe this ratio increased in alignment with the physiological exfoliation times of these teeth.

At the end of the follow-up period > 24 months in our study, the clinical success rates for HA, FC, and FS agents were 100%, 90%, and 94.7%, respectively, with no statistically significant difference found among the groups. In studies with a 24-month follow-up period comparing FC with FS, analyses conducted by Smaïl-Faugeron et al. [39] did not report statistically significant differences between the materials, and similarly, in studies with a follow-up duration exceeding 18 months, analyses conducted by Tewari et al. [40] also did not indicate any statistically significant differences between the materials. These results are in line with our study. Additionally, in their study, Huth et al. [32] reported that despite no statistically significant superiority was found after a 3-year follow-up, FS treatments exhibited clinically better as compared to FC treatments, similar to our study.

In our previous study [29], although no significant differences were observed among the materials in terms of clinical success rates at the 12th-month follow-up, a substantial decrease in clinical findings and a notable increase in clinical success rates were observed in all three groups from the 12th month onward until the control period of retrospective analysis. The increase in clinical success rates was determined to be 12.5% in the HA and FC groups, and 7.9% in the FS group. Additionally, in clinical success rates, only the HA group exhibited increases of 1.2% during the period from the 1st-month to the 1st-year follow-up, moreover, 12.5% from the 1st-year follow-up extending to the last retrospectively analyzed period. In contrast, the other two control groups showed a decrease from the 1st-month to the 1st-year follow-up [29].

Tenderness to percussion was the most prevalent clinical findings throughout the entire first-year follow-up periods [29]; in our retrospective analysis, this observation was only noted in the FS group, with no similar finding in the HA and FC groups, and mobility was the most common clinical finding (2 FC, 1 FS), and none in the HA group. Smaïl-Faugeron et al. [39] published their analysis in the Cochrane Library and clinical pain was reported in 4 studies with 2 cases for FC and none for FS at 24th-month. In Sushynski et al. study [33], 4 teeth in the DFC group showed abscess in a two-year period. Sonmez et al. [34] reported swelling findings in 2 teeth in the FC group in a two-year study, and in the study by Petel et al. [38], sinus tract formation was observed in 2 teeth in the FC group. In our RCT with a 1-year follow-up [29] and the present retrospective study, no teeth showed spontaneous, nocturnal pain and/or swelling.

The HA, FC, and FS groups demonstrated 81.5%, 80%, and 78.9% radiographic success rates, respectively, with no statistically significant differences found among the groups. When comparing the 1st-month with > 24 months retrospective control period, the radiographic success rate had an increase of 3.1% in the HA group, while a decrease of 7.5% and 16% in the FC and FS groups, respectively. The radiographic success rates were found to be lower than the clinical success rates, throughout all follow-up periods in our RCT [29] and the present retrospective study, excluding the 1st month for the FC and FS groups. We considered teeth with advanced physiological root resorption evident on radiographs as successful since they did not exhibit radiographic and clinical findings, similar to the study by Erdem et al. [36].

In our previous RCT [29], internal root resorption was the most prevalent radiographic finding throughout the entire first-year follow-up periods, with external root resorption being the second most common radiographic finding. In our long-term retrospective examination, internal root resorption was the most common pathological radiographic finding, with furcal radiolucency being the second most prevalent; external root resorption was detected in only 2 teeth.

In our initial one-year follow-up RCT [29], PCO was not observed; during our extended retrospective analysis, PCO became a prevalent radiographic feature. In line with studies by Sonmez et al. [34], Noorollahian [35], and Nematollahi et al. [37], we did not categorize PCO as a radiographic failure. Similarly, Erdem et al. [36] reported PCO as the most non-pathologic, and internal root resorption as the most pathologic radiographic findings in their second-year follow-up study. On the other hand, Nematollahi et al. [37] reported a statistically significantly higher PCO in the FC group compared to teeth treated with MTA after a two-year follow-up. In our study, however, the FC group emerged as the group with the least observed PCO, and no statistically significant difference was detected among the materials used.

Numerous studies in the literature have investigated the efficacy of HA on hDPSCs [28, 41–43]. Umemura et al. [41] declared that in HA-containing hDPSCs cultures, dentin sialophosphoprotein (DSPP) and dentin matrix acidic phosphoprotein 1 (DMP-1) mRNA levels significantly increased over 24 h, serving as odontoblastic differentiation markers, while HA also elevated DSPP and DMP-1 protein levels, leading to the conclusion that HA stimulates hDPSCs towards odontoblastic differentiation. When cultured with HA for one week, hDPSCs exhibited a concentration-dependent increase in alkaline phosphatase (ALP) protein levels, a mineralization indicator, with similar results obtained when measuring ALP amounts, suggesting that HA directs hDPSCs mineralization. In their in vitro study investigating the efficacy of HA on pulp cells isolated from 10 third molars, Chen et al. [42] stated that dental pulp cells treated with 2.0 mg/ml high molecular weight HA showed an early increase in ALP activity on the 3rd day, and additionally observed that dental pulp cells treated with HA for 3 days exhibited more and larger mineralized nodules compared to control group cells. They also reported the upregulation of crucial mineralization-associated molecules such as tissue-nonspecific alkaline phosphatase (ALP), bone morphogenetic protein 7 (BMP7), and type XV collagen (Col15A1) through the HA-CD44 pathway in vitro. Schmidt et al. [43] recently investigated the efficacy of HA molecules with low, medium, and high molecular weights on hDPSCs, and revealed that while long-term culture of hDPSCs with HA slightly influenced their characteristics positively, it preserved their stem cell characteristics and differentiation potentials. They also reported that none of the HA molecules they tested exhibited cytotoxic effects on hDPSCs. According to studies investigating the efficacy of HA on hDPSCs, there are few in vivo and in vitro animal studies in the literature examining the efficacy of HA on dental pulp [44, 45]. Sasaki and Kawamata-Kido [44] concluded that, as a result of the direct pulp capping treatment they conducted with HA on the molars of female Sprague-Dawley rats, HA may influence the environment appropriate for reparative dentin formation over existing dentin walls by means of the differentiation of undifferentiated mesenchymal cells into odontoblast-like cells. In their in vitro study, Bogović et al. [45] examined the efficiency of pulp capping preparations consisting of HA, CH, and dentine adhesive on cells isolated from Sprague-Dawley rats. They reported that HA was the most efficient and the least toxic material for direct pulp capping and could be advised for such treatments.

In light of the numerous experimental and animal studies mentioned above demonstrating the tissue regeneration supported by HA in dental pulp tissue or at the cellular level, our study also demonstrated promising results for HA in long-term success of human primary molar pulpotomy treatments. Mahfouz et al. [46] also reported that the success of their pulpotomy treatments using a 1:1 mixture of HA gel and ZOE cement as pulpotomy material in human primary molars, compared to FC material, was comparable based on the 12-month follow-up. However, the long-term follow-up results regarding the success of the materials have not been published to the authors’ knowledge.

In our retrospective analysis, the teeth treated with HA maintained clinically healthy until the physiological exfoliation time, while radiographically, there was a decrease in the number of findings, particularly internal root resorption. These results could highlight the significant healing potential of HA on human primary molar pulps in the long term. However, further histological studies and comprehensive, long-term follow-up human studies are needed to better understand the effect and mechanism of HA on dental pulp of human primary or permanent teeth.

One of the limitations of the study could be considered the difficulty in tracking data in long-term studies. In our previous RCT [29], only 2 dropouts were observed within the 1-year follow-up periods, whereas in our retrospective study with a maximum follow-up period of 30 months, data for 12 previously treated teeth (3 children) were not available both clinically and radiographically. One factor contributing to the data deficiency is the lack of regular follow-up appointments due to non-compliance among families. Additionally, the interference of the Covid-19 pandemic during these follow-up periods may have further impacted data collection. Furthermore, since the HA evaluated for long-term success in the present study is a commercially available product (Gengigel®Teething, Ricerfarma, Italy) with a 0.5% HA content, there is no data regarding the long-term effects of different HA concentrations on human primary molar pulp tissue in our study. To achieve the optimal effect of HA on pulp tissue, further research is needed to determine the ideal concentration and application time of HA.

## Conclusions

Within the limitations of this study, 0.5% hyaluronic acid gel exhibited comparable success rates to formocresol and ferric sulphate materials spanning over 24 months examinations, and given the limitations associated with the latter materials, as well as convenient accessibility and applicability of hyaluronic acid, it may be recommended as a promising alternative medicament for pulpotomy treatments of primary molars.

## Data Availability

The datasets used and/or analysed during the current study are available from the corresponding author on reasonable request.

## Abbreviations

FC: Formocresol
DFC: Diluted formocresol
GA: Glutaraldehyde
FS: Ferric sulphate
CH: Calcium hydroxide
MTA: Mineral trioxide aggregate
BD: Biodentine
ZOE: Zinc oxide eugenol
NaOCl: Sodium hypochlorite
AAPD: The American Academy of Pediatric Dentistry
HA: Hyaluronic acid
ECM: Extracellular matrix
hDPSC: Human dental pulp stem cell
RCT: Randomized clinical trial
SSC: Stainless steel crown
PCO: Pulp canal obliteration
EAPD: The European Academy of Paediatric Dentistry
DSPP: Dentin sialophosphoprotein
DMP-1: Dentin matrix acidic phosphoprotein 1
ALP: Alkaline phosphatase
BMP7: Bone morphogenetic protein 7
Col15A1: Type XV collagen
